# Supplementary material for: The vulnerability assessment and obstacle factor analysis of urban agglomeration along the Yellow River in China from the perspective of production-living-ecological space
Source: PLoS One. 2024 Apr 5;19(4):e0299729. doi: 10.1371/journal.pone.0299729 (PMC10997113; doi:10.1371/journal.pone.0299729)
Supplement: Attachment — Submitted filename: Response to Reviewers.docx [file pone.0299729.s002.docx]

Response to Reviewer 1 Comments on **“The vulnerability assessment and obstacle factor analysis of urban agglomeration along the Yellow River in China from the perspective of production-living-ecological space”**

First of all, we would like to thank you for your valuable comments. All the suggestions are of great importance for us to improve the manuscript quality. Therefore, we have revised our manuscript according to the suggestions and made point-to-point responses to the reviewer’s comments, and correcting the writing language and grammar in the new manuscript.

**Point 1:** The font format in Figure 1 needs to be modified, and please carefully review the entire article format.

**Response 1:** Thank you very much and agree with your question. We have modified the font and size of Figure 1 and carefully checked the format of the full text to meet the requirements of the Plosone journal.

Vulnerability index

| Year | Guanzhong Plain urban agglomeration | Central Plains urban agglomeration | Shandong Peninsula urban agglomeration | Jinzhong urban agglomeration | Hubao and Eyu urban agglomeration | Lanxi urban agglomeration | Ningxia urban agglomeration |
| --- | --- | --- | --- | --- | --- | --- | --- |
| 2001 | 0.27 | 0.41 | 0.47 | 0.28 | 0.28 | 0.26 | 0.31 |
| 2002 | 0.28 | 0.41 | 0.47 | 0.29 | 0.29 | 0.26 | 0.3 |
| 2003 | 0.27 | 0.41 | 0.49 | 0.27 | 0.28 | 0.26 | 0.29 |
| 2004 | 0.26 | 0.41 | 0.49 | 0.28 | 0.29 | 0.26 | 0.28 |
| 2005 | 0.26 | 0.41 | 0.5 | 0.27 | 0.31 | 0.25 | 0.26 |
| 2006 | 0.27 | 0.41 | 0.49 | 0.27 | 0.32 | 0.26 | 0.26 |
| 2007 | 0.27 | 0.42 | 0.48 | 0.28 | 0.34 | 0.26 | 0.27 |
| 2008 | 0.27 | 0.43 | 0.49 | 0.27 | 0.35 | 0.25 | 0.28 |
| 2009 | 0.27 | 0.44 | 0.51 | 0.28 | 0.36 | 0.26 | 0.27 |
| 2010 | 0.27 | 0.42 | 0.48 | 0.27 | 0.36 | 0.25 | 0.25 |
| 2011 | 0.28 | 0.42 | 0.46 | 0.29 | 0.34 | 0.25 | 0.24 |
| 2012 | 0.29 | 0.42 | 0.48 | 0.3 | 0.37 | 0.28 | 0.26 |
| 2013 | 0.29 | 0.42 | 0.47 | 0.3 | 0.36 | 0.27 | 0.26 |
| 2014 | 0.28 | 0.41 | 0.47 | 0.29 | 0.34 | 0.27 | 0.26 |
| 2015 | 0.3 | 0.44 | 0.49 | 0.3 | 0.36 | 0.29 | 0.28 |
| 2016 | 0.29 | 0.43 | 0.48 | 0.29 | 0.35 | 0.29 | 0.27 |
| 2017 | 0.3 | 0.44 | 0.48 | 0.3 | 0.35 | 0.3 | 0.27 |
| 2018 | 0.3 | 0.45 | 0.49 | 0.3 | 0.35 | 0.31 | 0.27 |
| 2019 | 0.31 | 0.45 | 0.46 | 0.28 | 0.32 | 0.31 | 0.26 |
| 2020 | 0.3 | 0.45 | 0.46 | 0.28 | 0.33 | 0.3 | 0.25 |

**Point 2:**  Please add the innovations and contributions of this paper in the literature review section to reflect the value of this paper.

**Response 2:** Thank you very much and agree with your question. We have clarified the innovations and academic contributions of this article, as follows:

Line111-127: The innovations and contributions of this article are: (1) Taking the seven urban agglomerations along the Yellow River as the research object, a better understanding of the integrity and systematicity of the Yellow River urban agglomerations will help promote integration and coordination among cities. The research results have certain reference significance for urban management planning and sustainable development in other similar regions around the world. (2) On the basis of existing research, we establish an index system for evaluating vulnerability that is more comprehensive and efficiently evaluates the vulnerability of the urban agglomerations along the Yellow River between 2001 and 2020. From the perspective of production-living-ecological space, the original assessment index system is synthesized, and new indicators are creatively introduced in conjunction with the real conditions of the research region. (3) We analyze the vulnerability assessment results from the perspective of the overall vulnerability and spatial variability of the urban agglomeration. Furthermore, the obstacle factor model was used to identify the influencing factors, and then the evolution trend of the obstacle factors was analyzed. Based on these, targeted management measures are proposed for the urban agglomeration along the Yellow River.

**Point 3:** In the first paragraph of 4.2.2, " In 2018, among the 32 provincial-level administrative regions in China, Anhui Province ranked 22nd in terms of per capita GDP, making it an economically underdeveloped province. However, in recent years, the GDP growth rate of Anhui Province has been relatively fast, and the total GDP has jumped to the tenth place in the country in 2022." The two economic concepts of per capita GDP and total GDP are used to explain the enhancement of Anhui Province's economic strength, which I think are not comparable, and it is suggested to unify the concepts.

**Response 3:** Thank you for your suggestions. We have carefully checked the full text and found no issues you mentioned. The research area of this article does not involve Anhui Province. At the same time, in the comprehensive evaluation index system for the vulnerability of the urban agglomeration along the Yellow River constructed in this article, we measure the quality of living space through per capita GDP, and measure the quality of ecological space through energy consumption per 10,000 yuan of GDP, industrial wastewater discharge, and carbon emissions. There is no comparison between per capita GDP and total GDP as you pointed out. Therefore, we made no modifications here.

**Point 4:** In the policy recommendations, there are no specific suggestions based on the results of the paper.

**Response 4：**Thank you very much and agree with your question. In the manuscripts submitted earlier, we have put forward specific suggestions for each urban agglomeration for the results studied in this article, as follows:

Line733-788: Based on the above conclusions, we propose several suggestions to enhance the quality of urban development along the Yellow River urban agglomeration.

(1) Formulate a three-level development strategy. Based on the vulnerability of each urban agglomeration, local governments should formulate urban agglomeration development plans based on local conditions. Highly vulnerable urban agglomerations should implement a green circular economy model, moderately vulnerable cities need to focus on optimizing industrial structure and upgrading production technology, and low-vulnerability urban agglomerations should accelerate opening up and industrial regional cooperation. In particular, the urban agglomeration of Shandong Peninsula ought to initiate ecological restoration initiatives and cultivate a varied energy framework. The creation of a circular economy model and bolstering the growth of international commerce scale should be the priorities of the Central Plains urban agglomeration. The metropolitan agglomerations of Yu, Baotou, and Hubei should increase their investments in research and technology, introduce more professional talents, and support the modernization of production methods and the optimization of the industrial structure. The urban agglomerations of Jinzhong, Guanzhong Plain, Lanxi, and Ningxia along the Yellow River are expected to foster coordinated development and strengthen regional collaboration.

(2) Pay attention to ecological and environmental protection. Local governments should coordinate and reconcile the contradiction between ecology and development and adhere to sustainable development. For central cities with high vulnerability, some functions of the city should be distributed to surrounding cities to alleviate the pressure on the urban ecological environment; while surrounding cities should strengthen urban greening construction and promote regional cooperation to form an urban ecosystem and realize inter-city collaborative development. At the same time, local governments should establish ecological restoration projects. For upstream urban agglomerations such as Ningxia urban agglomeration, the construction of green ecological corridors should be accelerated to ensure the integrity and stability of the ecosystem in the Yellow River Basin. For urban agglomerations along the lower reaches of the Yellow River such as the Central Plains urban agglomeration, it is necessary to Continue to promote pollution prevention and control, carbon reduction and emission reduction, and enhance the ecological barrier function of the Yellow River Basin.

(3) Develop domestic and foreign trade. Due to locational constraints, the level of opening-up of urban agglomerations along the Yellow River is relatively effective. In this regard, governments at all levels should increase infrastructure investment and construction, establish a high-quality transportation network throughout the Yellow River Basin, and promote urban interconnection. At the same time, we should take Shandong Peninsula as the window of regional foreign trade, and vigorously develop export-oriented industries and carry out import and export business. And provide preferential policies to attract regional investment, improve the level of foreign trade development.

(4) Properly plan and manage the tourism industry. Regional governments should give full play to the leading role of the tourism industry. Based on the regional Yellow River cultural advantages, relevant policies have been introduced to develop tourism into a regional advantageous industry. Urban agglomerations with high tourism and leisure vulnerability should optimize tourism resource management, enhance tourism resource utilization efficiency, and develop low-carbon tourism products and projects to reduce energy consumption in the tourism industry. For urban agglomerations with a low level of tourism industry development, they should promote technological upgrading and structural optimization and upgrading of the tourism industry by accelerating the introduction of tourism professionals and promoting talent training and information exchange. At the same time, each urban agglomeration should also vigorously promote urban tourism cooperation. This is the main way to promote the complementary advantages of the tourism industry in the upper, middle and lower reaches of the Yellow River Basin and narrow regional differences. The ultimate goal is to promote coordinated regional development.

**Point 5:** It is suggested to add the discussion part to make the structure of the article more complete.

**Response 5：**In the previously submitted manuscript, we have added a discussion section to compare this article with previous research to highlight the scientific nature and marginal contribution of this research, as follows:

Line637-704: Based on the above research results, we compare this article with previous research results to illustrate the scientific nature and marginal contribution of this article.

Overall, the urban agglomeration along the Yellow River has a remarkable three-level gradient differential in terms of vulnerability. This characteristic is the same as earlier research, and closely corresponds to the overall vulnerability gradient differentiation of Chinese urban agglomerations [13]. This demonstrates that, as a whole, the degree of vulnerability of the urban agglomeration along the Yellow River examined in this research is congruent with the real circumstances in China. Because the gradient of vulnerability at all levels is generally consistent with previous research on urban areas in the upper, middle, and lower ranges of the Yellow River, it suggests that the study is scientific. On the other hand, the total vulnerability of the urban agglomeration along the Yellow River indicates an increasing trend, based on the specific value of the obstacle degree. The percentage of cities with high susceptibility rises, while the percentage of cities with moderate or high vulnerability stays constant. This is consistent with previous research conclusions on urban agglomerations in the Yangtze River Basin [34,35], Pearl River Basin [36] and other regions, but is contrary to research conclusions in northeast China [37], central Yunnan [38], Zhangjiakou [39] and other regions of China. It is evident that the vulnerability of the urban agglomeration along the Yellow River has a particular influencing mechanism.

Urban agglomeration is a coupled system of economy, society and environment. Prior research on the Yellow River Basin has mostly concentrated on environmental and economic issues [40-43]. The findings indicate that the primary determinants of the urban agglomeration's growth along the Yellow River are resource availability, ecological environment, and economic development. The research conclusions of this article have a high degree of fit with it. However, existing studies lack the perspective of living space quality. Although there are studies on social and people's livelihood aspects, they are more focused on policy support and lack research on urban tourism and leisure functions [44,45]. It can be seen that the existing research on the quality of urban development in the Yellow River Basin is incomplete. Therefore, this paper constructs a multi-dimensional index system to comprehensively evaluate the evolutionary characteristics of vulnerability barrier factors in the urban agglomeration along the Yellow River. Compared with existing evaluation methods, this index system is more systematic and scientific.

Contrary to earlier research, the study's findings indicate that the influence of leisure and tourism activities on the vulnerability of urban agglomerations along the Yellow River has grown dramatically and is especially significant. We proceed from an economic perspective based on supply and demand effects. In terms of demand, with the continuous development of China's economy and the continuous increase in national income, the demand for tourism is becoming increasingly strong. This has led to a continuous increase in the number of tourists per region, which has put forward higher-level requirements for the development of tourism in urban agglomerations along the Yellow River; At the same time, as China's economy shifts from high-speed development to high-quality development, the country has attached great importance to the protection of the Yellow River culture and the development of the tourism industry since 2020. Higher requirements have been placed on the average tourism income of urban agglomerations along the Yellow River, which has led to the increasingly prominent importance of tourism and leisure functions in the development of urban vulnerability. In terms of supply, urban agglomerations along the Yellow River mainly focused on the development of primary and tertiary industries in the past, with the tertiary industry accounting for a relatively low proportion. The tourism supply was seriously insufficient and difficult to meet social needs. Although the industrial structure has improved in recent years and the tertiary industry has developed, the development level of the tourism industry is not high, mostly at the expense of consuming resources and damaging the environment. The average number of tourists per area and the average tourism income are not high, which is extremely detrimental to the high-quality development of the city and increases the vulnerability of the urban agglomeration along the Yellow River.

In summary, the marginal contributions of this article are as follows: This article improves the existing indicator system and conducts a scientific analysis of the overall vulnerability and obstacle factor evolution of the urban agglomeration along the Yellow River. The high-quality development level of urban agglomerations along the Yellow River is studied not only from economic and environmental aspects, but also from social aspects. It was found that the tertiary industry, specifically tourism and leisure functions, is particularly important to urban vulnerability. Theoretically, this article enriches previous research on the Yellow River Basin and provides a new perspective on tourism and leisure research. In practice, the research results can guide the planning and management of urban agglomerations along the Yellow River, and provide reference for their sustainable development.

That’s all the responses.

We appreciate your suggestions sincerely.

Your efforts make our manuscript better.
